# Syphilis

**Published:** 1894-11

**Authors:** W. H. Whitslar

**Affiliations:** Cleveland, O.


### THE DENTAL REGISTER.

VOL. XLVIII.] NOVEMBER, 1894. [No. 11.

### COMMUNICATIONS.

### Syphilis.

AN AUTOBIOGRAPHICAL SKETCH.

BY W. H. WHITSLAR, M.D., D.D.S., CLEVELAND, O.

Read before the Northern Ohio Dental Society, June, 1894.

After being called the disease of the French, German, Poles, Turks, Spaniards, Neapolitans, and others, in which each nation tried to have another stand as sponsor for me, I received my name in the sunny clime of Italy. I was already hundreds of years old and my friends were scattered all over the world. Previous to this my guardians had failed to give me a name, although I am the daughter of Prostitution.

I was named after a herdsman of King Alkithous, Syphilis by name, who, having offended Apollo, the god vested his vengeance by giving me to him, at least, Fracastor, in a Latin poem in 1530 A. D., gave this history to the world.

The word syphilis descends in a direct line from the Greek, "with" and "love," the companion of love, meaning that the disease is transmitted by intimate relations.

The history of my career has been one of contention. I have lived in all parts of the world and visited all nations.

From the fact that I have appeared in different forms of disguise, I have been unrecognized by many, until I threw off the mantle, and the flaming signs of my virulence became cognizable. Even now the dress I wear may have different colors. I am partial to a red or copper color, and these fringed with blue or gray dominate my dress.

These colors may be evidences of my presence and liability to remain for a time, for I have a peculiar habit which in man is a virtue, in me a curse, namely, perseverence. I form intimate acquaintances and never leave them, although at times our lines are separated somewhat, because my light is kept under a bushel. By men I am introduced to their beautiful wives, and even become a part and parcel of their children. I am truly sorrowful to kill these poor little innocents, very often before they are born or soon after, but it is only the logical sequence of that persevering habit of adhering to those who seek my company, and fulfilling the old adage that the sins of the fathers shall be visited upon their children.

Misery loves company. My life has been one full of misery. I have seen thousands of people die and nations cursed in my presence, whilst I have survived. Though I have been said to be five thousand years old, I at times have tender feelings and need nursing. I have myriads of nurses. As a rule they are unfit to give me comfort, because they try to get rid of me. Perhaps this is because I am repulsive to many. My life is repugnant to me also, although I live in freedom in kings' palaces, and have seared the names of dignitaries of church and monastery, monks, nuns, etc., as well as to degrade the poor slave. My home is constantly being moved from one place to another. My personality is an enigma. Some authorities claim that I am a poison existing in the blood which I infect. Others devise that I am an individual germ capable of propagating my own species. This is not definitely settled. However, Lustgarten in 1884 described an S shaped bacillus found in tissue and discharges of syphilitic ulcers, but he was unable to cultivate it. It has been found in the blood by other observers. That I do poison men, I cannot deny. That I can race with unmeasured speed in flowing currents of blood and secretions is my delight. As yet no man has captured and pinned me to the walls of his dark museums. In these catacombs traces of my wrath may be seen. It is a fact, which may be recalled that in the Stone age my existence then is now proven by the marks of exostoses that I left upon the bones of man. Teeth also bear characteristic deformities. Broca, Ollier, Parrot and Virchow, have witnessed these results.

I am therefore prehistoric. Men have even suspected that I have existed since man was created.

Undisputed evidences chronicle my presence in China 2,637 years B.C., by the writings of an emperor, Hoan-Ti, who lived at that time. My cradle seems to have been rocked in India. The Aryans, a Sanscrit-speaking people of India, whose ancestors came from Central Asia, peopled Persia, Palestine, Arabia and Egypt, caused my virus finally to spread over Europe. I have visited the islands of the sea and also appeared in America before it was discovered by Columbus. Evidences of Peruvian subjects of this disease before Europeans made use of iron are well known.

Further history of my existence would require pages of print to complete it.

I will now mention two of my most prominent characteristics.

Most flowers have perfumes. Some are noted for their delicate odors, others are not so pleasant. Unfortunately, I am like the flowers with the disagreeable odor. It is said that once the smell from my breath penetrates the Schneiderian membrane of man it leaves a lasting remembrance.

Secondly. I have a monstrous appetite. It amounts to gluttony. I feed upon the vitals of men and women alike. Their blood I render thin, the red corpuscles are dismembered and their coloring matter assist in the syphilides. I feed on brains, oh, what a delicate dish! Many noted men have contributed theirs to appease my appetite. My greed is well described by E. L. Keyes, in the following language: "There is no organ or tissue of the body through which it may not manifest its presence by symptoms, or upon which it may not exercise its power. The lymphatic glands all over the body may suffer, some habitually more than others. The skin from crown to sole, the nails, the hair (the teeth in inherited disease), and the mucous membranes, especially through the natural orifices, have their peculiar affections due to syphilis. The eye and the testicle do not escape, and each and every viscus is liable to be invaded as are all the tissues, connective, fibrous, muscular, cartilage, bone, brain, nerve, and vessel. Not only this, but the all-embracing arms of general syphilis include the functions as well, any of which may be disordered by it, and each and all of the special senses may be perverted or destroyed including the sexual appetite. The symptoms of all the forms of local, special, or general paralysis of motion or sensation may be occasioned by syphilis. Finally, the intellect may succumb. Acute and chronic mania, dementia, lunacy, idiocy, all the above and many more form the catagory of symptoms comprehended under the one term Syphilis."

It is my first essay to destroy the shameful parts, and indeed this is the first salutation to my master, who bought me with a price, and now he becomes *my* slave.

Inherited syphilis has no chancre. Chancre is a term given to a syphilitic primary sore. Although a misnomer, the name is retained by syphilographers to distinguish it from chancre, chancroid, a similar sore having a soft base, whilst the chancre has an indurated base. There are other characteristic differences and the principal one is the edge of the sore, chancre, edge sloping, chancroid undermined.

Chancre has a period of incubation of ten days to two weeks. Chancroid has no incubation. The secretion of the chancroid is purulent and auto-inoculable. Lindnan has inoculated himself 2,700 times at last report with chancroidal secretion. Chancre may have no secretion, or if so, it is serous and scanty. The chancre (syphilitic) is not transferable to animals as is chancroid, and appears but once in a lifetime. Zeigol says he has seen reinfection.

Chancre is followed by a train of constitutional ebullitions. Ricord states that an individual is never free from syphilis if it is acquired. Chancroid is not accompanied by any constitutional syphilitic infection.

The appearance of chancres on extra-genital parts is limited to a small percentage. That they are contagious is evidenced by many facts. Prof. Tarnowski, of St. Petersburg, relates a case of a woman who came under his observation who contaminated 300 men in ten months by primary transmission. Think of the consequences! Yet, the disease is rarely fatal except in the viscera form. "Gonorrhœa," says Keyes, speaking of its consequences, "sends more to the grave than syphilis."

Syphilis may be divided into three stages, i. e.:

*Primary.*—The chancre, lasting from four to six weeks, found principally on the genitals; it may be found on the lips, in the mouth, on the tonsils, or in the pharynx. Dentists should be careful not to inoculate their fingers by wounds from an instrument laden with the poison from a syphilitic sore, or they may do the same by holding an instrument with the lips, fissures in which would permit the poison to penetrate to the deeper tissues. In dental operations the chief source of danger is in the mucous patch. This secretes the intensely contagious substance which may adhere to the instruments, napkins, rubber dam, dental floss, plaster, etc. The disease may be transmitted by tooth transplantation.

The *Secondary* stage may last from two months to a year. It is contra-distinguished from the third stage, by its symmetrical effects. The first and second stages affect the cutaneous parts of the body. The third stage involves the connective tissues.

The cutaneous eruptions are called syphilides (sif-il-eeds), and the following may occur: erythema, papules, vesicles, pustules, bullæ, and gummata and tubercles.

Now, you may ask me "What are some of the manifestations in the mouth of man?"

Naturally our attention reverts to the teeth. Inherited disease, vitiating the blood of the mother during pregnancy, causes a lack of nutrition and proper calcification of, principally, the central incisors and molars of the permanent set of teeth. The incisors are most affected, because of their smaller lobes, these being deficient, especially the central lobe, the mesial and distal ones are melted down, as it were, to conform to the central one, and consequently the mesial and distal angles are rounded, giving the rounded or "peg-shaped" tooth as described by Mr. Jonathan Hutchinson. The central lobe, easily disintegrated, favors the "notched" appearance. A study of the time of development of these will enable one to readily understand why these teeth are affected more than others. Syphilis affects only the permanent set of teeth, as above indicated. Oakly Coles reports peg-shaped teeth in a temporary set. The general debility may reduce the quality of structure in the temporary set. The same expressions of debility will dwarf the alveolar process. Syphilis affects the jaw bones as well as other bones. It must be understood that acquired syphilis may result from syphilitic mothers or nurses, and the full gamut of the disease may be run in the young child, In this event the arrest of development of the jaws may continue so as to have a relative disproportion in the size of the bones compared to the teeth. "Arrest of development of the jaws, however, the result of constitutional diseases, to produce dental deformities, must occur prior to the sixth year."— *Talbot.*

The diagnosis of congenital syphilis is not entirely dependent upon the teeth, for some syphilitics have good teeth.

*Tertiary* syphilis may manifest itself as—

1. Gummatous tumor—nodular diffuse.
2. Periostitis.
3. Ulcers (mostly deep, result in caries and necrosis).

When the third form is found it is usually in the median line either at the junction of soft and hard palate or over the hard palate itself. It may also be found just behind the upper incisor teeth. Mucous patches of the mouth, in either second or third stages, are quite characteristic. They consist of bluish-white elevations of mucous membrane; their favorite location is on the tonsils and soft palate. These should not be confounded with tubercular ulceration. The dentist must beware of mucous patches. Their secretion is syphilitic poison. Unless these or other ulcerations of the mouth are present, saliva or other physiological secretions cannot produce syphilis by inoculation. Profeta, of Palermo, and Diday inoculated saliva without success. Secretions from chancre, mucous patches, and secondary cutaneous or mucous lesion yielding a discharge, and of syphilitic blood taken from patient with eruption, will yield an indurated chancre. Syphilis is sure to follow, even though the chancre is excised a short time after its presence is known, because the virus is readily absorbed. I would conclude this sketch with the language of Lennox Brown.

"The history of the case, the post-cervicle glandular enlargement, absence of sympathetic induration of the parotid, submaxillary or anterior cervicle glands, the comparative freedom from pain, and above all, its amenity to appropriate remedies will distinguish this disease from cancer, the only malady with which it is confounded."

